# Supplementary material for: Dynalign II: common secondary structure prediction for RNA homologs with domain insertions
Source: Nucleic Acids Res. 2014 Nov 21;42(22):13939–48. doi: 10.1093/nar/gku1172 (PMC4267632; doi:10.1093/nar/gku1172)
Supplement: SUPPLEMENTARY DATA [file supp_gku1172_nar-02021-z-2014-File012.zip › manual/javadoc/RNAstructure_java_drawing/source/proxy/DotPlotBackendProxyJNI.html]

DotPlotBackendProxyJNI


JavaScript is disabled on your browser.


- Overview
- Package
- Class
- Tree
- Index
- Help

- Prev Class
- Next Class

- Frames
- No Frames

- All Classes

- Summary:
- Nested |
- Field |
- Constr |
- Method

- Detail:
- Field |
- Constr |
- Method


RNAstructure\_java\_drawing.source.proxy

## Class DotPlotBackendProxyJNI

- java.lang.Object
- - RNAstructure\_java\_drawing.source.proxy.DotPlotBackendProxyJNI

- ---

    

  ```
   class DotPlotBackendProxyJNI
  extends Object
  ```

- - ### Constructor Summary

    Constructors

    Constructor and Description

    `DotPlotBackendProxyJNI()`
  - ### Method Summary

    Methods

    | Modifier and Type | Method and Description |
    | --- | --- |
    | `static void` | `delete_DotPlotBackend(long jarg1)` |
    | `static String` | `DotPlotBackend_getBounds(long jarg1, DotPlotBackend jarg1_)` |
    | `static String` | `DotPlotBackend_getColors(long jarg1, DotPlotBackend jarg1_)` |
    | `static double` | `DotPlotBackend_getCurrentMax(long jarg1, DotPlotBackend jarg1_)` |
    | `static double` | `DotPlotBackend_getCurrentMin(long jarg1, DotPlotBackend jarg1_)` |
    | `static double` | `DotPlotBackend_getDefaultMax(long jarg1, DotPlotBackend jarg1_)` |
    | `static double` | `DotPlotBackend_getDefaultMin(long jarg1, DotPlotBackend jarg1_)` |
    | `static String` | `DotPlotBackend_getDotData(long jarg1, DotPlotBackend jarg1_, int jarg2, int jarg3)` |
    | `static String` | `DotPlotBackend_getDotDataByLocation(long jarg1, DotPlotBackend jarg1_, int jarg2, int jarg3, double jarg4)` |
    | `static String` | `DotPlotBackend_getGridLine(long jarg1, DotPlotBackend jarg1_, int jarg2)` |
    | `static String` | `DotPlotBackend_getLegendData(long jarg1, DotPlotBackend jarg1_)` |
    | `static int` | `DotPlotBackend_getLength(long jarg1, DotPlotBackend jarg1_)` |
    | `static boolean` | `DotPlotBackend_readData(long jarg1, DotPlotBackend jarg1_, String jarg2, int jarg3)` |
    | `static void` | `DotPlotBackend_setColors(long jarg1, DotPlotBackend jarg1_, int jarg2)` |
    | `static void` | `DotPlotBackend_setRange(long jarg1, DotPlotBackend jarg1_, double jarg2, double jarg3)` |
    | `static void` | `DotPlotBackend_writePostscriptFile(long jarg1, DotPlotBackend jarg1_, String jarg2)` |
    | `static String` | `DotPlotBackend_writeStructuresFile(long jarg1, DotPlotBackend jarg1_, String jarg2, String jarg3)` |
    | `static void` | `DotPlotBackend_writeSVGFile(long jarg1, DotPlotBackend jarg1_, String jarg2)` |
    | `static void` | `DotPlotBackend_writeTextFile(long jarg1, DotPlotBackend jarg1_, String jarg2)` |
    | `static long` | `new_DotPlotBackend()` |

    - ### Methods inherited from class java.lang.Object

      `clone, equals, finalize, getClass, hashCode, notify, notifyAll, toString, wait, wait, wait`

- - ### Constructor Detail


    - #### DotPlotBackendProxyJNI

      ```
      DotPlotBackendProxyJNI()
      ```
  - ### Method Detail


    - #### delete\_DotPlotBackend

      ```
      public static final void delete_DotPlotBackend(long jarg1)
      ```


    - #### DotPlotBackend\_getBounds

      ```
      public static final String DotPlotBackend_getBounds(long jarg1,
                                    DotPlotBackend jarg1_)
      ```


    - #### DotPlotBackend\_getColors

      ```
      public static final String DotPlotBackend_getColors(long jarg1,
                                    DotPlotBackend jarg1_)
      ```


    - #### DotPlotBackend\_getCurrentMax

      ```
      public static final double DotPlotBackend_getCurrentMax(long jarg1,
                                        DotPlotBackend jarg1_)
      ```


    - #### DotPlotBackend\_getCurrentMin

      ```
      public static final double DotPlotBackend_getCurrentMin(long jarg1,
                                        DotPlotBackend jarg1_)
      ```


    - #### DotPlotBackend\_getDefaultMax

      ```
      public static final double DotPlotBackend_getDefaultMax(long jarg1,
                                        DotPlotBackend jarg1_)
      ```


    - #### DotPlotBackend\_getDefaultMin

      ```
      public static final double DotPlotBackend_getDefaultMin(long jarg1,
                                        DotPlotBackend jarg1_)
      ```


    - #### DotPlotBackend\_getDotData

      ```
      public static final String DotPlotBackend_getDotData(long jarg1,
                                     DotPlotBackend jarg1_,
                                     int jarg2,
                                     int jarg3)
      ```


    - #### DotPlotBackend\_getDotDataByLocation

      ```
      public static final String DotPlotBackend_getDotDataByLocation(long jarg1,
                                               DotPlotBackend jarg1_,
                                               int jarg2,
                                               int jarg3,
                                               double jarg4)
      ```


    - #### DotPlotBackend\_getGridLine

      ```
      public static final String DotPlotBackend_getGridLine(long jarg1,
                                      DotPlotBackend jarg1_,
                                      int jarg2)
      ```


    - #### DotPlotBackend\_getLegendData

      ```
      public static final String DotPlotBackend_getLegendData(long jarg1,
                                        DotPlotBackend jarg1_)
      ```


    - #### DotPlotBackend\_getLength

      ```
      public static final int DotPlotBackend_getLength(long jarg1,
                                 DotPlotBackend jarg1_)
      ```


    - #### DotPlotBackend\_readData

      ```
      public static final boolean DotPlotBackend_readData(long jarg1,
                                    DotPlotBackend jarg1_,
                                    String jarg2,
                                    int jarg3)
      ```


    - #### DotPlotBackend\_setColors

      ```
      public static final void DotPlotBackend_setColors(long jarg1,
                                  DotPlotBackend jarg1_,
                                  int jarg2)
      ```


    - #### DotPlotBackend\_setRange

      ```
      public static final void DotPlotBackend_setRange(long jarg1,
                                 DotPlotBackend jarg1_,
                                 double jarg2,
                                 double jarg3)
      ```


    - #### DotPlotBackend\_writePostscriptFile

      ```
      public static final void DotPlotBackend_writePostscriptFile(long jarg1,
                                            DotPlotBackend jarg1_,
                                            String jarg2)
      ```


    - #### DotPlotBackend\_writeStructuresFile

      ```
      public static final String DotPlotBackend_writeStructuresFile(long jarg1,
                                              DotPlotBackend jarg1_,
                                              String jarg2,
                                              String jarg3)
      ```


    - #### DotPlotBackend\_writeSVGFile

      ```
      public static final void DotPlotBackend_writeSVGFile(long jarg1,
                                     DotPlotBackend jarg1_,
                                     String jarg2)
      ```


    - #### DotPlotBackend\_writeTextFile

      ```
      public static final void DotPlotBackend_writeTextFile(long jarg1,
                                      DotPlotBackend jarg1_,
                                      String jarg2)
      ```


    - #### new\_DotPlotBackend

      ```
      public static final long new_DotPlotBackend()
      ```


- Overview
- Package
- Class
- Tree
- Index
- Help

- Prev Class
- Next Class

- Frames
- No Frames

- All Classes

- Summary:
- Nested |
- Field |
- Constr |
- Method

- Detail:
- Field |
- Constr |
- Method
